# Stay Home! Stay Safe! First Post-Discharge Cardiologic Evaluation of Low-Risk–Low-BNP Heart Failure Patients in COVID-19 Era

**DOI:** 10.3390/jcm10102126

**Published:** 2021-05-14

**Authors:** Nadia Aspromonte, Luigi Cappannoli, Pietro Scicchitano, Francesco Massari, Ivan Pantano, Massimo Massetti, Filippo Crea, Roberto Valle

**Affiliations:** 1 Department of Cardiovascular Sciences, Fondazione Policlinico Universitario Agostino Gemelli, IRCCS, 00168 Rome, Italy; massimo.massetti@policlinicogemelli.it (M.M.); filippo.crea@policlinicogemelli.it (F.C.); 2 Institute of Cardiology, Catholic University of the Sacred Heart, 00168 Rome, Italy; luigi.cappannoli@gmail.com; 3 Cardiology Section, F. Perinei Hospital, 70022 Altamura (BA), Italy; piero.sc@hotmail.it (P.S.); franco_massari@libero.it (F.M.); 4 Cardiology Department, Madonna della Navicella Hospital, 30015 Chioggia (VE), Italy; ivan.pantano@aulss3.veneto.it (I.P.); roberto.valle@aulss3.veneto.it (R.V.)

**Keywords:** B-type natriuretic peptide, heart failure, management, prognosis

## Abstract

Background. The COVID-19 pandemic has had a deep impact on periodic outpatient evaluations. The aim of this study was to evaluate the impact of low brain natriuretic peptide (BNP) values in predicting adverse events in heart failure (HF) patients in order to evaluate implications for safe delay of outpatient visits. Methods. This was a retrospective study. One-thousand patients (mean age: 72 ± 10 years, 561 women) with HF and BNP values <250 pg/mL at discharge were included. A 6-month follow-up was performed. The primary endpoint was a combination of deaths and readmissions for HF within 6-month after discharge. Results. At 6-month follow-up, 104 events (10.4%) were recorded (65 HF readmissions and 39 all-cause deaths). Univariate Cox analysis identified as significant predictors of outcome were age (*p* < 0.001, hazard ratio [HR] = 1.044), creatinine (*p* = 0.001, HR = 1.411), and BNP (*p* < 0.001, HR = 1.010). Multivariate Cox regression confirmed that BNP (*p* < 0.001, HR = 1.009), creatinine (*p* = 0.016, HR = 1.247), and age (*p* = 0.013, HR = 1.027) were independent predictors of events in HF patients with BNP values <250 pg/mL at discharge. Patients with BNP values >100 pg/mL and creatinine >1.0 mg/dL showed increased events rates (from 4.3% to 19.0%) as compared to those with lower values (*p* < 0.000, HR = 4.014). Conclusions. Low pre-discharge BNP levels were associated with low rates of cardiovascular events in HF patients, independently of the frequency of follow-up.

## 1. Introduction

Since the beginning of 2020, coronavirus disease (COVID)-19 has spread worldwide, affecting the cardiovascular system both directly and indirectly [1,2] and forcing the rapid and radical transformation of our health systems during the last year. There are still many concerns about the risk of re-opening while the disease is still being transmitted.

During this period, the general attitude has been to prioritize caring for COVID-19 patients with critical and time-sensitive clinical presentation. Indeed, patients not affected by COVID-19 are facing too many difficulties in terms of the management of both acute emergencies and chronic conditions such as heart failure (HF) [3]. Urgent, innovative approaches should be managed to ensure access to medical care, avoiding risk of exposure.

The COVID-19 pandemic reveals the need for more accurate diagnostic information able to provide better health decisions in order to avoid unnecessary access to Emergency Departments (ED) and, in parallel, timely care of illnesses other than COVID-19.

Plasma B-type Natriuretic Peptide (BNP) is the strongest prognostic predictor in HF. Low pre-discharge BNP values allow identifying a cohort of hospitalized patients with low event rates during the following six-month follow up [4,5].

The use of plasma BNP values for risk stratification of HF patients may improve the personalization of treatments [6].

The aim of this study was to evaluate a possible mechanism for stratifying the HF population in order to evaluate the group of such individuals who may benefit from early admission to outpatient clinics and/or ED or those whose evaluation might be postponed due to being considered low-risk.

## 2. Materials and Methods

We reviewed the HF outpatient databases of three Italian centers in order to identify low-risk patients who could be safely managed, deferring at six months after discharge. These centers were representative of the Italian scenario (North: Chioggia, Venice; Centre: Rome; South: Altamura, Bari).

This study was approved by our institutional ethics committee with a waiver for informed consent because of the retrospective nature of the study.

One thousand patients with HF and BNP values at discharge <250 pg/mL were retrospectively identified through the HF database records of three centers according to a common protocol [7].

We excluded those with (1) concomitant unstable angina or myocardial infarction, (2) pending revascularization procedures, or (3) terminal malignancy with less than 12 months of life expectancy.

HF was defined as follows: (1) symptoms of HF according to the criteria commonly accepted in the literature [8], namely the presence of two major criteria or one major criterion plus two minor criteria according to the Framingham score; (2) exacerbation of HF symptoms with at least one NYHA class deterioration; (3) evidence of left ventricular systolic and/or diastolic dysfunction at echocardiographic examination; (4) BNP levels > 100 pg/mL on admission.

Patients were considered “stable” in the case of (1) subjective improvement in NYHA functional class with no orthopnea; (2) systolic blood pressure 90–120 mmHg; (3) heart rate < 100 bpm; (4) ambient air blood oxygen saturation >90%; and (5) diuresis > 1000 mL/day [7].

A 6-month follow-up was performed either by outpatient visits, computerized chart review, and/or telephone call. The primary clinical endpoint selected was a combination of deaths and readmissions to hospital for heart failure within 6 months after discharge.

Blood samples were analyzed upon admission with the BNP Triage method (Biosite Diagnostics, La Jolla, CA, USA), an immunofluorescence method that is performed in situ on whole blood and that requires a plasma volume of 250 μL with a detection limit as low as 10 pg/mL.

Echocardiographic examinations were performed on admission and were reviewed in a blinded fashion with respect to BNP values. Left ventricular systolic dysfunction was defined as a left ventricular ejection fraction (LVEF) < 50% [9]. Left ventricular diastolic dysfunction was defined according to previously validated criteria [10].

### Statistical Analysis

Data are expressed as mean ± standard deviation (continuous data) and numbers (percentages). A *p*-value < 0.05 was considered statistically significant. Means were compared using one-way ANOVA with Tukey’s test. Cox proportional hazards regression models were employed to assess the relation of clinical variables, BNP and creatinine levels, and echocardiographic parameters with the incidence of the primary combined endpoint (death from any causes and readmissions for heart failure) during 6 months after discharge. Cut-off values were selected using the area under the curve (AUC) index based on the receiver operating characteristic (ROC). Kaplan–Meier curves were generated to compare the occurrence of events in relation to BNP levels. Analyses were performed using SPSS software for Mac (Chicago, Illinois).

## 3. Results

### 3.1. Baseline Characteristics

Baseline characteristics of the study population are summarized in Table 1. The patient population included 561 women and 439 men, aged 72 ± 10 years. The main HF etiology was ischaemic heart disease (IHD: 38%). The percentages of preserved, mid-range and reduced LVEF were 35, 32, and 33% of the overall population, respectively. Prescription of evidence-based drugs was as follows: ACE inhibitors, 78.3%; sartans, 12.1%; beta-blockers, 60.9%; Mineralocorticoid Receptor Antagonists, 51.5%. At discharge, most patients reported subjective improvement in mean NYHA class, with 80% in NYHA class I-II. At discharge, NYHA class was 2.1 ± 0.6 and BNP value 110 ± 73 pg/mL.

### 3.2. Clinical Events

During the 6-month follow-up, 104 events (10.4%) were recorded (65 readmissions for HF decompensation and 39 all-cause deaths). Seven-hundred three patients were evaluated at 6-month follow-up, while 297 patients were lost. Univariate Cox analysis identified three variables as significant predictors of outcome: age (*p* < 0.001, HR = 1.044, CI = 1.022–1.067), creatinine (*p* = 0.001, HR = 1.411, CI = 1.196–1.1.666), and BNP (*p* < 0.001, HR = 1.010, CI = 1.007–1.013). The hazard ratios for the other variables were not significant. In particular, LVEF was not correlated with the event-free survival (*p* = 0.763, HR = 0.997). Specifically, cardiologic follow-up did not influence the final predictive model (*p* = 0.060, HR = 0.683). Multivariate Cox regression confirmed that BNP (*p* < 0.001, HR = 1.009, CI = 1.006–1.012), creatinine (*p* = 0.016, HR = 1.247, CI = 1.042–1.492), and age (*p* = 0.013, HR = 1.027, CI = 1.006–1.049) were independent predictors of events in HF patients with BNP values <250 pg/mL at discharge.

Therefore, we tried to differentiate our population according to both BNP and creatinine values (Table 1).

**Figure 1:**
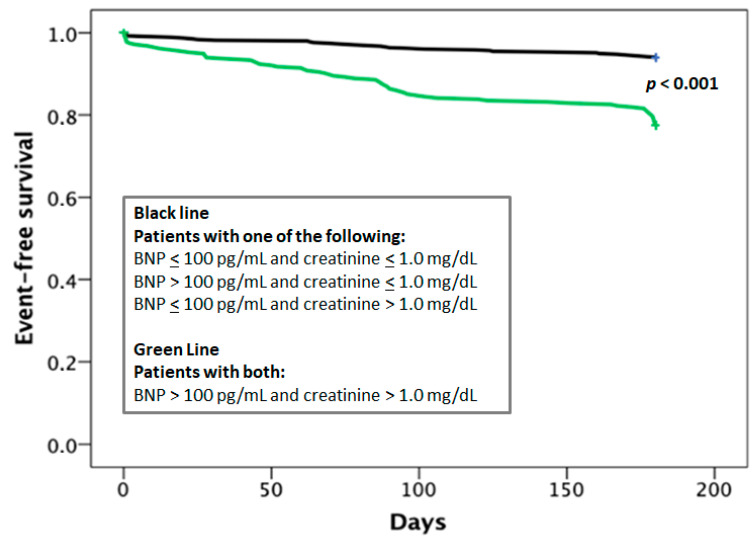
Kaplan–Meier curves comparing the occurrence of events in relation to combined BNP and creatinine cut-off levels.

In particular, we identified a cohort of patients at very low-risk when BNP values were <100 pg/mL and creatinine <1.0 mg/dL as the corresponding ROC analysis showed optimal predictive values (96% and 90% respectively). Event rates increased from 4.3% to 19.0% in patients with either no or one (*n* = 632) versus both of these variables above the cut-off values (*n* = 368) (Cox regression: *p* < 0.000, HR = 4.014) (Figure 1).

## 4. Discussion

Our study confirms previous observations that patients with low (<250 pg/mL) BNP values are at very low risk of events within six months after discharge [11,12,13]. Among this cohort of low-risk patients, a combination of plasma BNP and serum creatinine allows us to separate patients with residual risk from those at very low risk (<5%) of events.

In the COVID-19 era, early identification of HF patients who could be safely managed “far from” the hospital has become an urgent need. The results of our study outlined the possibility to promote the assessment of individuals with HF and low plasma BNP and serum creatinine values status without the involvement of hospital visit at least after six months. The modern management of HF patients before COVID-19 has been based on the rational use of health resources to optimize diagnostic and therapeutic procedures. The COVID-19 pandemic enforced this managerial route in order to provide early admission to care for patients at higher risk for adverse events. Therefore, the new health plan should differentiate individuals who might be evaluated later in the follow-up period. Low BNP values predicted favorable outcomes in patients admitted for HF [4]. Maisel et al. [14] reported a 9% event rate at 3-month follow-up in patients with admission of BNP levels <200 pg/mL. Our study suggests that pre-discharge BNP values <250 pg/mL may allow identifying patients who have been stabilized by medical treatment and need delayed follow-up. Indeed, BNP predicted a very low number of events in our low-risk population. Despite their advanced age, most of our patients had few comorbidities, with a high proportion showing preserved left ventricular function and isolated diastolic HF. All these features have been reported to correlate with lower adverse events rate, thus suggesting that patients may be discharged safely with minimal follow-up protocols [15,16,17,18,19,20].

An unexpected finding of our study is that cardiologic follow up did not predict readmission-free survival rate when BNP at discharge was <250 pg/mL. The risk of an unfavorable outcome in our population was independent of a close cardiologic follow up. Multidisciplinary strategies for the management of patients with HF are effective in reducing HF hospitalizations [21]. In particular, programs involving specialized follow up by a multidisciplinary team reduce both mortality and all-cause hospitalizations [22]. The nationwide survey on acute HF in Italy [23] reported a re-hospitalization rate of 38.1% at 6-month follow up, while data from Medicare beneficiaries who were hospitalized for HF [24] demonstrated a 30-day readmission rate of 21.3%, in patients who underwent one-week follow-up after discharge. A more precise risk stratification at discharge for HF patients would therefore probably help to identify only those patients requiring more stringent follow up.

Our results are in line with the literature. Schou et al. [25] demonstrated that HF patients who were hemodynamically stable and on optimal medical therapy could delay access to the outpatient healthcare system. A meta-analysis from McQuade et al. [26] outlined the need for reaching lower values in BNP/NT-proBNP predischarge of patients hospitalized for acute HF in order to predict the exact impact of these parameters on the overall mortality rate of the patients.

The main limitation of our study is its non-randomized, retrospective nature. In addition, the low event rate resulted in low statistical power. However, our results are consistent with data currently available in the literature for patients with low pre-discharge BNP values [27,28,29]. Another limitation is the need for considering the predictive value of NT-proBNP in patients on sacubitril-valsartan therapy. Further studies are needed in order to evaluate this specific biomarker in the context of low-risk HF patients.

## 5. Conclusions

Low pre-discharge BNP levels are associated with low rates of cardiovascular events in HF patients, independently from the frequency of cardiology follow-up visits. BNP levels appear to be useful in reducing the number of patients who will mostly benefit from specific cardiologic follow-up during the first 6 months after hospital discharge. BNP values may help to reduce the number of unnecessary visits above all during the COVID-19 era, thus resulting in a lower risk of exposure to contagion both for patients and for health care operators.

## Figures and Tables

**Table 1:** Clinical and demographic characteristics of the study patients.

Parameters	Total (*n* = 1000)	BNP ≤ 100 pg/mL and crea ≤ 1 mg/dL (*n* = 161) Group a	BNP > 100 pg/mL and crea ≤ 1 mg/dL or BNP ≤ 100 pg/mL and crea > 1 mg/dL (*n* = 471) Group b	BNP > 100 pg/mL and crea > 1 mg/dL (*n* = 368) Group c
Age (years)	72 ± 10	67 ± 12	72 ± 9a	73 ± 10a
Male gender (%)	54	56	55	52
NYHA class	2.1 ± 0.6	2.0 ± 0.5	2.1 ± 0.6	2.2 ± 0.6a
LVEF (%)	46 ± 12	48 ± 11	46 ± 11	44 ± 13a, b
BNP (pg/mL)	110 ± 73	45 ± 27	84 ± 65a	173 ± 44a, b
Creatinine (mg/dL)	1.3 ± 0.7	0.8 ± 0.1	1.3 ± 0.7a	1.5 ± 0.8a, b
Ischemic heart disease	38	36	37	40
Atrial fibrillation	26	24	24	28
Diabetes mellitus (%)	25	25	26	27
Events (*n*, %)				
Death	39 (3.9)	1 (0.6)	13 (2.8)	25 (6.8)
Readmission for HF	65 (6.5)	6 (3.7)	14 (3.0)	45 (12.2)
Total	104 (10.4)	7 (4.3)	27 (4.7)	70 (19.0)

## Data Availability

Data will be available on request by contacting the corresponding author.
